# Ketones for Post-exercise Recovery: Potential Applications and Mechanisms

**DOI:** 10.3389/fphys.2020.613648

**Published:** 2021-01-26

**Authors:** Latt Shahril Mansor, Geoffrey Hubert Woo

**Affiliations:** Health Via Modern Nutrition Inc. (H.V.M.N.), San Francisco, CA, United States

**Keywords:** exercise, recovery, exogenous ketones, ketosis, ketogenic diet, d-beta-hydroxybutyrate

## Abstract

Ketogenic diet has been introduced in therapeutic areas for more than a century, but the role of ketones in exercise performance has only been explored in the past decade. One of the main reasons that allows the investigation of the role of ketones in exercise performance is the emergence of exogenous ketones, allowing athletes to achieve the state of ketosis acutely, and independent of their metabolic states. While there are mixed results showing either exogenous ketones improve exercise performance or no effect, the mechanisms of action are still being heavily researched. Moreover, these early data from exercise physiology studies suggested that exogenous ketones may play a more prominent role in post-exercise recovery, leading to a more pronounced cumulative impact over subsequent exercise performance. This review will look at existing evidence on the role of ketones in recovery and attempt to identify the current best practices and potential mechanisms that drive improved recovery.

## Introduction

**Figure 1:**
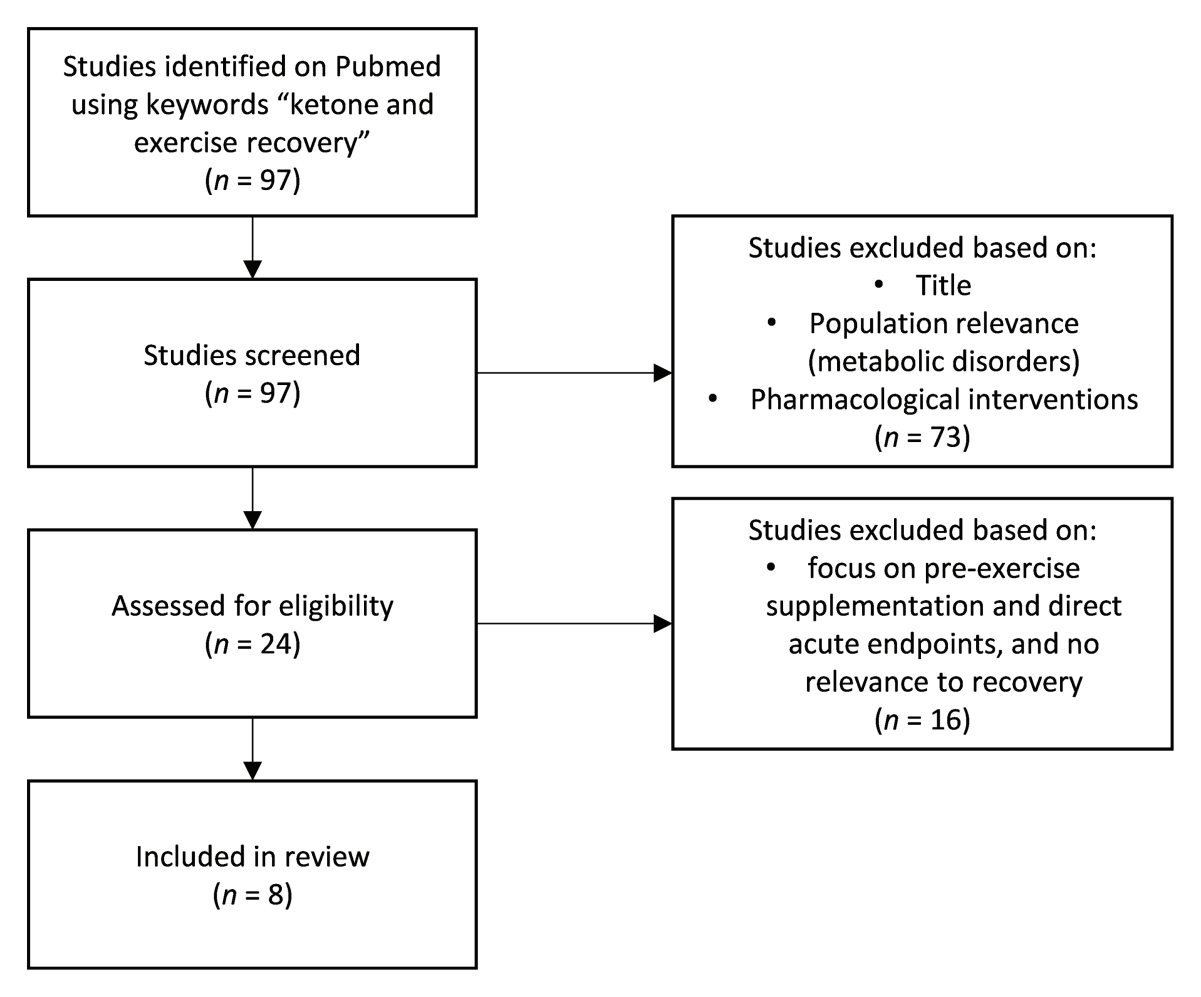
Flowchart of studies collection.

The impact of ketosis, induced endogenously *via* a ketogenic diet (Prado et al., 2011; Huang et al., 2018; Ma et al., 2018a,b) or exogenously through exogenous ketones (Cox et al., 2016; Evans and Egan, 2018; Evans et al., 2019; Poffe et al., 2020a,b) on physical performance is an active, quickly evolving area of inquiry. Most studies to date have focused on probing a potential ergogenic impact of acute exogenous ketone ingestion pre-exercise. The corpus is mixed to date and potential utility is dependent on context specificity, on form of exogenous ketone, dosing, co-supplementation, and protocol. However, recent work investigating the role of ketones for post-exercise recovery suggests another more durable and clear application for ketones in sport performance. We identified and screened all relevant studies using the key phrase “ketone and exercise recovery” on PubMed (Figure 1) to identify the current bodies of research in post-exercise recovery involving ketones. Although some early animal studies showed that endogenous ketones, achieved by feeding animals with ketogenic diet, may be protective against oxidative damage and improve recovery (Huang et al., 2018; Ma et al., 2018a), there are also concerns that the lack of carbohydrates in the body may hinder performance, especially in the higher intensity bouts of exercise (Cox et al., 2016). As per Randle cycle, the increase in fatty acid oxidation decreases the glucose metabolic pathway (Randle et al., 1965), and therefore, ketogenic diet upregulating fatty acid metabolism may inhibit glycolytic rates and, subsequently, the capacity to produce ATP independent of oxygen availability. This is especially crucial for explosive and high intensity anaerobic exercises. This has always been the concern of researchers about utilizing ketogenic diet for performance or recovery. However, with the emergence of exogenous ketones, athletes may be able to increase their blood β-hydroxybutyrate (BHB) concentrations and be in ketosis, regardless of the diet they are on as well as being able to stack different substrates with ketones to optimize its effects in performance and recovery.

This review aims to elucidate the findings in animal and clinical work on the impact of ketones for post-exercise recovery, the potential mechanisms of action, and the metabolic interplay with other recovery-relevant metabolic pathways. We also examine the strengths and limitations between endogenous and exogenous ketosis in a post-exercise recovery indication.

## Ketogenic Diet and Post-Exercise Recovery

The goal of the ketogenic diet is to achieve optimal nutritional ketosis, which is defined within the range of 0.5 up to 3.0mM of blood BHB but could also potentially reach up to 5–8mM BHB, depending on the keto-adaptability of the individual (Volek and Phinney, 2011; Poff et al., 2020). The earliest studies on ketones were done on ketogenic diet, with investigating if endogenous ketones from ketogenesis have an ergogenic effect on exercise performance and subsequently during recovery post exercise. In animal studies, fat- and keto-adapted mice on ketogenic diet for 8weeks (Table 1) showed enhanced performance without aggravated muscle injury while exerting potential protective effect on organ injury caused by exercise (Ma et al., 2018b). The authors suggested that this may be attributed to the increased efficiency in fat and ketone metabolism in the ketogenic diet group of mice as Ma et al. showed decreased BHB, non-esterified fatty acids (NEFA) and triglycerides during exercise while lipase was unchanged, indicating a more proficient manner at which these metabolites were mobilized and utilized in the ketogenic diet group. The same group of researchers, in another study, showed that an 8-week ketogenic diet not only enhanced exercise performance, but also attenuated muscle damage caused by exhaustive exercise as well. On top of that, the group of mice on ketogenic diet had improved fatigue recovery following exhaustive exercise, which may have contributed to the overall enhanced performance (Huang et al., 2018). The authors also investigate further to determine the potential mechanisms and additional markers that may indicate how ketogenic diet may help with performance and recovery. Plasma interleukin (IL)-6 was previously reported to increase over 100-fold after strenuous exercise (Suzuki et al., 1999, 2000, 2003), and this excessive exercise-induced IL-6 secretion may induce muscle damage and be detrimental to athlete’s health and performance (Suzuki et al., 1999, 2003). Exercise-induced IL-6 was also shown to stimulate lipolysis both in the intramuscular triglycerides’ pool and adipocytes (Path et al., 2001; Carey et al., 2006; Ma et al., 2018a). However, direct mechanism of action connecting ketone to these biomarker changes has not yet been determined. Given the overarching function of IL-6 on lipid metabolism and strenuous exercise, it has become a relevant biomarker to illustrate the relationship between ketogenic diet and exercise. Animal models showed that ketogenic diet may prevent muscle damage in specific muscle fiber types by mitigating excessive IL-6 synthesis and secretion during exercise (Ma et al., 2018a). Another biomarker that is often associated with muscle damage and interference with muscle energy imbalance is creatine kinase (CK). Ketogenic diet may play an important role in decreasing oxidative damage markers such CK and lactate dehydrogenase (LDH) caused by exhaustive exercise (Ma et al., 2018b).

**Table 1:** List of studies that investigated effects of ketogenic diet and ketones on post-exercise recovery.

**Human studies**					
**Study**	**Reference**	**Population description**	**Sample size**	**Ketone supplement**	**Performance recovery outcome**
1	Prado et al., 2011	Trained cyclists	13 males	Ketogenic diet + keto analogues associated with amino acids (KAAA) vs. control (lactose)	KAAA diminished exercise-induced hyperammonemia
**Animal and *in vitro* studies**					
**Study**	**Reference**	**Population description**	**Sample size**	**Ketone supplement**	**Performance recovery outcome**
1	Takahashi et al., 2019	*In vitro* epitrochlearis muscles	N/A	Glucose (8mM) + insulin (60 μU/ml) + 1/2/4mM sodium BHB vs. glucose (8mM) insulin (60 μU/ml) only	Four millimolar BHB had a significant positive effect on glycogen repletion in epitrochlearis muscle at 120min after exercise while 2mM of BHB showed a tendency to increase the glycogen level
2	Huang et al., 2018	C57BL/6J mice	35 males	Eight-week ketogenic diet vs. chow diet	Exercise-induced injury biomarkers improved by KD, accompanied by accelerated recovery with increased locomotion after exhaustive exercise
3	Ma et al., 2018a	C57BL/6J mice	35 males	Eight-week ketogenic diet vs. chow diet	KD mitigated excessive IL-6 synthesis and secretion during exercise, contributing to the prevention of muscle damage
4	Ma et al., 2018b	C57BL/6J mice	35 males	Eight-week ketogenic diet vs. chow diet	Showed a potential preventive effect on organ injury caused by acute exercise but KD failed to exert protection from muscle injury

Several studies have demonstrated that increased ammonemia occurs during various types of exercise (Wagenmakers et al., 1990; Carvalho-Peixoto et al., 2007; Bassini-Cameron et al., 2008). In fact, this was confirmed that during prolonged submaximal exercise, an increase in ammonemia (0.160mmol/L) has been observed in various studies (Graham and MacLean, 1992; Bassini-Cameron et al., 2008). It has been suggested that ammonia promotes both central and peripheral fatigue and that better control of ammonia production will improve exercise performance (Banister and Cameron, 1990). The production of ammonia is increased as adenosine monophosphate (AMP) deamination and catabolism of amino acids occur during exercise and these processes are driven by the intensity and duration of the exercise (Wilkinson et al., 2010; Prado et al., 2011). While substrates, such as glucose and fatty acids, are metabolized during exercise to produce energy, amino acids undergo reversible processes, such as deamination or transamination, producing keto acids *via* the release of the amino group, which subsequently contributes to the formation of ammonia and ammonemia in the blood. Since these reactions are reversible, then providing keto analogues could potentially reduce blood ammonia concentration by forming amino acids with the amino groups (Prado et al., 2011). Prado et al. showed that the exercise-related rise in blood ammonia concentration was attenuated in the group of participants on a keto acid and amino acid supplementation in combination with ketogenic diet, while sustaining the decreased blood ammonia concentration during the recovery period. Although the authors did not pinpoint the exact mechanisms that took place, it was speculated that the ketones and amino acid supplementation replenish the Kreb cycle intermediates *via* anaplerosis, hence decreasing the rate of amino acid catabolism, or it could be the chelation of ammonia by the keto analogues (Prado et al., 2011). Additionally, ammonia level during exercise was correlated with blood urea nitrogen (BUN), the final product of blood ammonia. Huang et al. used BUN as a kidney injury marker, indicator of exercise tolerance as well as a protein degradation marker. In their study, BUN was significantly lower in ketogenic diet groups after 24-h post exercise compared to the chow group (Huang et al., 2018). This shows the presence of ketones as well as the fat-adapted metabolic capacity may improve central and peripheral fatigue post exercise, suggesting that ketones may be a potential strategy for better recovery and long-term performance enhancement. However, further investigations are warranted to determine the exact mechanism of action that connects lipid metabolism and nervous system-driven fatigue.

## Experimental Gap Using Ketogenic Diet for Recovery

The aim of an optimal post-exercise recovery strategy is to ensure that the body adapts to the external stimuli in the form of improving metabolic efficiency and physiological adaptation for speed, strength, and endurance, without incurring excessive oxidative damage and preventing injury from progressive overload training. Subsequently, due to these adaptation processes that interact with each other in the body, one will need less time to recover and return to the peak physical condition followed by improved performance over time. Carbohydrates and proteins have been long established as important substrates to aid glycogen recovery and muscle repair after exhaustive exercises (Zawadzki et al., 1992; Ivy et al., 2008). Therefore, by restricting one or both of these food groups in a recovery intervention may be counter-productive. This is the biggest disadvantage of the ketogenic diet or using endogenous ketones as a recovery strategy. The introduction of substantial glucose or excess protein into the diet will put the participants out of the optimal nutritional ketosis state and lower the blood ketone levels. Hence, this diminishes the benefits that ketones may provide during recovery. This dilemma can now be solved by using exogenous ketones as the acute rise in blood ketone concentrations caused by exogenous ketones is not inhibited by the ingestion of glucose nor the minor insulin spike that follows, allowing the additive effect of stacking multiple substrate types to enhance the ergogenic effects on recovery. It is certainly vital for blood BHB concentrations to reach a certain level before the benefits are observed and exogenous ketones can elevate blood BHB concentrations significantly in an acute setting. For example, a ketone monoester, R-β-hydroxybutyrate-R 1,3-butanediol monoester, has been shown in multiple studies to increase blood BHB concentrations up to 3mM and above within 60min of ingestion independent of the metabolic state of the individual (Cox et al., 2016; Myette-Cote et al., 2019; Stubbs et al., 2019; Poffe et al., 2020a,b).

## Exogenous Ketones in Recovery

The introduction of commercially available exogenous ketones enabled a research tool to probe a physiological state of ketosis in the presence of carbohydrate and protein consumption. This research paradigm allows a deeper understanding of the impact of ketones on metabolism and their interplay with other metabolic substrates. Three studies to date have focused on post-exercise recovery following ingestion of ketone monoester as the primary endpoint (Holdsworth et al., 2017; Vandoorne et al., 2017; Poffe et al., 2019). Although there are discrepancies between these studies, all three studies have found some form of benefits of ketone monoester, when taken with recommended macronutrients such as carbohydrate and protein after workout, and improved recovery (Table 2). Holdsworth et al. (2017) showed in subjects with a hyperglycemic clamp, holding glucose at 10mM, which is at a supraphysiological concentration, ketone monoester increased glucose uptake by 32% in the intervention arm compared with control. This was associated with a 2-fold increase in insulin concentration in the ketone monoester group compared with control group, indicating when glucose and ketones are present together after exercise, ketones may play a role in increasing insulin secretion (Beylot et al., 1986) and directly increasing activity of glucose transporter type 4 (GLUT4; Yu et al., 2016) to help cells take in more glucose for recovery and repair. This was evident as the muscle glycogen was 50% higher in the ketone group compared to the control group, explaining the upstream drivers that are increased insulin secretion and uptake of glucose. This is consistent with the *in vitro* work by Takahashi et al. (2019) that showed improved glycogen repletion after exercise by incubation mice epitrochlearis muscles with physiological concentrations of glucose, insulin and 4mM BHB. The question remains, does ketone increase insulin sensitivity and responsiveness *per se* or simply magnifying the effects of exercise has on both of these parameters as explained by Zorzano et al. (1986). While insulin has been conventionally associated with glucose regulation, it is a powerful regulator in metabolism that goes beyond regulating a single substrate. In fact, insulin plays a vital role in protein synthesis and anabolism, which is extremely important in skeletal muscles recovery following exercise to repair and adapt for improved performance over time (Fryburg et al., 1995; Timmerman et al., 2010).

**Table 2:** List of studies using ketone monoester (in combination with other substrates) as post-exercise recovery strategy.

Study	Reference	Population description	Sample size	Age (year)	Ketone supplement	Performance recovery outcome	Experimental period for recovery and blood BHB concentration
1	Holdsworth et al., 2017	Trained athletes	12 males	33 ± 6.5	Single dose of R-β-hydroxybutyrate-R 1,3-butanediol monoester (573mg/kg) + hyperglycemic clamp vs. control + hyperglycemic clamp vs. control + saline clamp	Ketone monoester and glucose clamp increased glucose uptake by 32% and muscle glycogen content by 50%	Average 3–5mM blood BHB for 120min
2	Vandoorne et al., 2017	Healthy adults	8 males	20–24	R-β-hydroxybutyrate-R 1,3-butanediol monoester (5mg/kg) (single dose) followed by 0.25g/kg BW/h (multiple dose) + CHO (1g/kg BW/h) and protein (0.3g/kg BW/h) vs. placebo + CHO (1g/kg BW/h) and protein (0.3g/kg BW/h) only	Ketone monoester with a standard post-exercise recovery beverage enhances the post-exercise activation of mTORC1	Average 3–5mM blood BHB for 270min
3	Poffe et al., 2019	Healthy adults	12 males	21.2 ± 2.9	R-β-hydroxybutyrate-R 1,3-butanediol monoester (25 g) + CHO (60.6 g) and protein (30 g) vs. placebo + CHO (60.6 g) and protein (30 g)	Sustainable training load in week 3 as well as power output in the final 30 min of a 2‐h standardized endurance session were 15% higher in ketone monoester than in control	2.6 ± 0.2 mM blood BHB within 30 minutes post exercise. Experiment lasted 3 weeks and there was no continuous monitoring available

In terms of protein resynthesis as part of recovery after exercise, Vandoorne et al. showed no glycogen increase in their study, perhaps due to a physiological oral carbohydrate intake as compared to the Holdsworth et al. study. However, Vandoorne et al. indicated that ketone monoester promotes mammalian target of rapamycin complex 1 (mTORC1) signaling in recovery following exercise. Two downstream targets of mTOR involved in protein synthesis, which are phosphorylation status of P70^S6k1^ at Thr^389^ (p-S6K1^Thr389^), and the percentage of 4E-BP1 in the phosphorylated γ-form (4E-BP1%γ) were measured and both were significantly increased in the ketone monoester group (Vandoorne et al., 2017). An especially important point is to note about the protocol of Vandoorne et al. is the inclusion of proteins together with carbohydrates and ketone monoester. As proven in their *in vitro* work, the combination of leucine and ketone bodies increased p-S6K1^Thr389^ ~ 6-fold and 4E-BP1%γ 2-fold vs. baseline, indicating the synergistic effects of leucine and ketone bodies in enhancing leucine-mediated muscle protein synthesis and potentially on a systemic level, amplifying the stimulation of exercise and supplementation on recovery. In addition, ketone bodies were previously shown to decrease proteolysis in starvation as it may suppress the rates of protein breakdown to cater for gluconeogenesis (Owen et al., 1967; Cahill, 2006). It has been shown that following intense exercise, energy balance *via* AMP-activated protein kinase (AMPK) sensing mechanism in skeletal muscles need to be restored before mTOR activation can take place to upregulate protein synthesis (Dreyer et al., 2006). Therefore, ketone monoester ingestion for recovery may provide the additional substrate that aids oxidative ATP generation and subsequently decreases AMPK activation to allow protein synthesis. This is in agreement with Thomsen et al. (2018) who suggested that BHB plays a vital role as potent anticatabolic agent in both systemic and skeletal muscle-specific levels, where the reduction of protein breakdown catalyzed by BHB overrides inhibition of synthesis. There are some studies that showed evidence that mTORC1 is also likely activated by a growth factor-independent movement of proteins to and from the lysosome, *via* resistance exercise-induced phosphorylation of the tumor suppressing gene, Tuberous Sclerosis Complex 2 (TSC2) (Watson and Baar, 2014). Other studies with other forms of ketone esters have shown similar results, where skeletal muscle atrophy and inflammation-induced catabolism were prevented by administration of acetoacetate diester in mice (Koutnik et al., 2019, 2020).

Much remains to be studied on the relationship between mTOR and different substrates in both systemic and local environments. Absence of mTOR in knockout mice models showed glucose intolerance and insulin resistance characterized by reduced glucose uptake in the muscle and reduced glycogen and lipid deposition in the liver under high fat diet condition (Guridi et al., 2016) while hyperactivation or sustained activation of mTOR has may contribute to hyperglycemia and insulin resistance *via* inhibition of insulin-induced Protein kinase B (AKT) phosphorylation, blocking glucose uptake in skeletal muscles (Williamson et al., 2015). This indicates the delicate control of mTOR, both in the precision in mTOR activation and sensitivity of its targets, is needed for optimal benefits in specific situations.

More recently, Poffe et al. (2019) published data indicating ketone monoester may play a role in preventing overreaching symptoms and even help athletes improve performance over the course of 3weeks after incorporating ketone monoester as part of their nutritional intervention for recovery. Compared to the previous publications on ketone monoester in recovery, this study extended the period of experiment to investigate the impact of ketone monoester, thus giving us a better understanding of how ketone monoester may help with recovery in the medium term and also the compounding effects over this period on performance. After all, the goal for an optimal recovery is to develop positive adaptation to improve speed, strength, and endurance. Despite symptoms of overreaching, which is the decrease in performance in the sprint tests, the ketone monoester group managed to improve their overall work output and endurance performance test by 15% compared to the control group, while reflected by the gradual increase in energy intake and expenditure in the former group (Poffe et al., 2019). However, similar to Vandoorne et al., Poffe et al. also did not see any difference in glycogen resynthesis rates in recovery following ingestion of ketone monoester as opposed to the findings by Holdsworth et al., suggesting that glycogen resynthesis may not be obligate in augmenting ketone-induced exercise recovery. One reason is that glucose was infused intravenously in Holdsworth et al. study, leading to higher blood glucose level and corresponding insulin secretion. However, the addition of protein as part of the post-exercise intervention in Vandoorne et al. and Poffe et al. may play a role as well. The activation of downstream targets of mTOR is significantly more pronounced when leucine and ketone bodies are present (Vandoorne et al., 2017), indicating that adding protein to the carbohydrate and ketone monoester supplementation for recovery will activate mTOR and upregulate protein synthesis. In addition, when mTORC1 is activated, S6K1 could directly phosphorylate Insulin receptor substrate 1 (IRS1; S307 and S636/S639) and promote its degradation, which subsequently blunts phosphoinositide 3-kinase (PI3K)-AKT activation and its downstream effects such as glucose uptake and glycogen accumulation (Laplante and Sabatini, 2012; Mao and Zhang, 2018). This suggests that the activation of mTORC1 *via* co-ingestion of protein and ketone monoester may inhibit the improved glycogen synthesis seen in previous studies, but not to the extent of being detrimental for recovery. After exhaustive exercise, muscle glycogen resynthesis needs of ~100mmol.kg^−1^ occur at an average rate of ~5mmol.kg^−1^.h^−1^, over the course of ~20h for complete recovery of glycogen stores and if given ample of carbohydrates at the beginning of recovery period, glycogen resynthesis rate may increase by 2–3mmol.kg^−1^.h^−1^ (Coyle, 1991; Burke et al., 2004). In fact, co-ingestion of carbohydrate with protein, when given at the beginning of recovery period, is effective to increase glycogen storage replenishment (Ivy et al., 2002). Since Poffe et al. did not see any significance difference in glycogen resynthesis rate between control and ketone monoester group, it may imply that the ketone monoester does not negatively impact glycogen resynthesis and augment the protein synthesis and repair processes.

Another potential benefits of exogenous ketones in recovery is to alleviate the sensation of fatigue. During exercise, levels of free tryptophan entering the brain are increased, causing an increase in 5-hydroxytryptamine (5-HT), a contributor to the sensation of fatigue (Watson et al., 2004; Hormoznejad et al., 2019). Ketone monoester was previously shown to decrease blood FFA (Cox et al., 2016). FFA competes for binding sites on albumin with tryptophan, and when levels of FFAs in the blood are decreased, free tryptophan concentration also decreases as more of tryptophan will be bound to albumin. This leaves less tryptophan to enter the brain and increase the sensation of fatigue as a result of exertion. Therefore, in addition to the physiological benefits to improve recovery, exogenous ketones may also provide avenue to improve mental strength and well-being, which ultimately may contribute to the improved adaptation and performance in the long-term.

Based on the data thus far, we can conclude that ketone monoester when taken together with glucose only, may increase insulin secretion, glucose uptake, and/or glycogen synthesis while ketone monoester taken with protein (especially containing leucine) and glucose, does not change glycogen synthesis but instead activate mTOR targets and upregulate leucine-mediated protein synthesis. In very specific cases, where recovery period is scarce (e.g., ultramarathon with minimal breaks between segments or military use cases that needs optimal endurance and output with minimal rest), ketone monoester and glucose administration may provide an option to ensure that glycogen is replenished in a shorter amount of time before the individual needs to expend energy for physical performance again. Otherwise, if given sufficient amount of carbohydrate that is over the threshold of maximal glycogen storage over 24h, glycogen stores will replete itself to baseline levels (Ivy et al., 2002; Burke et al., 2004). Therefore, for a better nutritional strategy for recovery, ketone monoester with glucose and protein would be a better option as one will still replenish their glycogen levels as seen in (Vandoorne et al., 2017), while benefitting from the repair and recovery of protein synthesis upregulation *via* activation of mTOR.

## Conclusion: Recovery Threshold, Potential Applications, and Mechanisms

The current data suggests that exogenous ketones taken after exercise in conjunction with carbohydrate and/or protein exerts an ergogenic effect in post-exercise recovery. Exogenous ketones also eliminate the disadvantage of ketogenic diet by allowing stacking of multiple substrates and supplements to enhance the speed and quality of recovery period. In the future, in addition to proteins and carbohydrates, other supplements, such as carnitine, creatine or even sodium bicarbonate, may be added to a recovery strategy to maximize what the body needs to adapt and grow. Recently, a study showed that sodium bicarbonate mitigate the mild acidosis caused by ketone monoester in athletes, thus unlocking the ergogenic potential of acute use of ketone monoester in performance enhancement (Poffe et al., 2020a). This suggests that sodium bicarbonate may be used to help the body to achieve optimal pH faster during recovery and provide the optimal environment for enzymes and hormones to begin the processes of recovery and repair. Further studies should be conducted to look at the role of pH in recovery especially in conjunction with ammonemia that accompanies exercise and drives fatigue.

**Figure 2:**
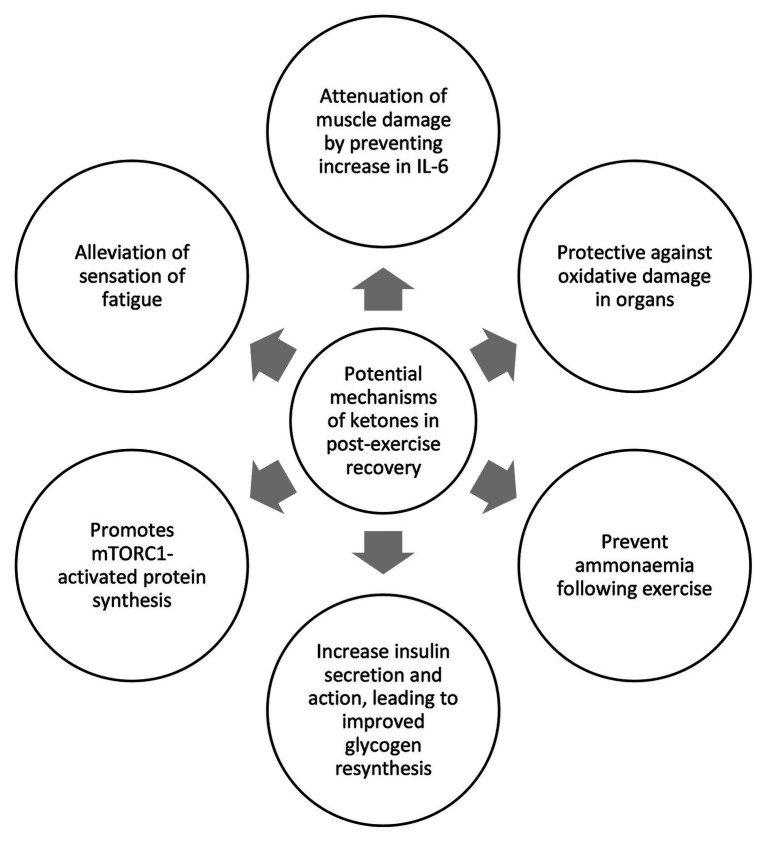
Summary of potential mechanisms of ketones in exercise recovery.

All three studies that used ketone monoester as part of their recovery strategy achieved between 2.5 and 5mM blood BHB following ketone monoester ingestion. Data further suggest a recovery benefit with a ketogenic diet alone, which typically yields blood BHB levels of 1–2mM. Therefore, we hypothesize the existence of a BHB recovery threshold, and we posit that this threshold is between 1–3 mM BHB. While the introduction of exogenous ketones in the field vastly increases the options and combinations of nutritional compositions to improve recovery, it is still too early to draw any definitive conclusion as the relevant studies are still few and far between. The summary of potential roles and mechanisms of ketones in post-exercise recovery based on the studies to date can be seen in Figure 2. The biggest limitation of this review paper is the lack of studies that examine the effect of ketones on post-exercise recovery as most of the studies have been focused on the acute ergogenic effects of ketones on exercise performance. We also acknowledge that post-exercise recovery studies are more difficult to be conducted as recovery covers multi-faceted aspects of physiology and it takes longer to measure the compounding effects of improved recovery. We hope that this review paper may spur more research groups to realize the potential of ketones in recovery and, thus, embark on more studies to illuminate the scientific community and the general public on the mechanisms involved. Further studies are needed to more precisely determine the threshold of blood BHB required for a positive recovery impact. Further work is also needed to determine if other forms of exogenous ketones, such as (R,S)-1,3-butanediol acetoacetate diester, 1,3-butanediol, or BHB salts or ketone precursors like caprylic acid and other medium-chain triglycerides compositions, are able to induce similar recovery benefit by raising BHB levels across the hypothesized threshold. Determining the existence and value of this hypothesized “ketone recovery threshold” will enable practitioners to deliver exogenous ketones more precisely in conjunction with optimal ratios of both carbohydrate and protein and their constituent sub-types.

## Author Contributions

LM was responsible for the literature research and manuscript preparation. GW involved in the manuscript preparation and support for the study. All authors contributed to the article and approved the submitted version.

### Conflict of Interest

LM is the Research Lead of Health Via Modern Nutrition Inc. (H.V.M.N.), which develops and commercializes products based on ketosis. GW is the founder and Executive Chairman of Health Via Modern Nutrition Inc. (H.V.M.N.). The authors declare that the results of the study are presented clearly, honestly, and without fabrication, falsification, or inappropriate data manipulation.
